# GAVCA Study: Randomized, Multicenter Trial to Evaluate the Quality of Ventricular Catheter Placement with a Mobile Health Assisted Guidance Technique

**DOI:** 10.1093/neuros/nyx420

**Published:** 2017-08-04

**Authors:** Ulrich-Wilhelm Thomale, Andreas Schaumann, Florian Stockhammer, Henrik Giese, Dhani Schuster, Stefanie Kästner, Alexander Sebastian Ahmadi, Manolis Polemikos, Hans-Christoph Bock, Leonie Gölz, Johannes Lemcke, Elvis Hermann, Martin U Schuhmann, Thomas Beez, Michael Fritsch, Berk Orakcioglu, Peter Vajkoczy, Veit Rohde, Georg Bohner

**Affiliations:** 1 Pediatric Neurosurgery and Department of Neurosurgery, Campus Virchow Klini-kum, Charité Universitätsmedizin Berlin, Berlin, Germany; 2 Pediatric Neurosurgery and Department of Neurosurgery, Universi-tätsmedizin Göttingen, Göttingen, Germany; 3 Department of Neurosurgery, Universitä-tsklinikum, Heidelberg, Heidelberg, Germany; 4 Department of Neurosurgery, Dietrich-Bonhoeffer-Klinikum, Neubrandenburg, Germa-ny; 5 Department of Neurosurgery, Klinikum Kassel, Kassel, Germany; 6 Department of Neurosurgery, Universitätsklinikum Düsseldorf, Düsseldorf, Germany; 7 Department of Neu-rosurgery, Medizinische Hochschule Hannover, Hannover, Germany; 8 Department of Neuro-surgery, Unfallkrankenhaus, Berlin Marzahn, Berlin, Germany; 9 Department of Neurosurgery, Universitätsklinikum Tübingen, Tübingen, Germany; 10 Department of Neuroradiology, Campus Virchow Klinikum, Universitätsmedizin Berlin, Berlin, Germany

**Keywords:** Hydrocephalus, Cerebrospinal fluid, Ventricular catheter guide, Mobile health, Shunt failure

## Abstract

**BACKGROUND:**

Freehand ventricular catheter placement may represent limited accuracy for the surgeon's intent to achieve primary optimal catheter position.

**OBJECTIVE:**

To investigate the accuracy of a ventricular catheter guide assisted by a simple mobile health application (mhealth app) in a multicenter, randomized, controlled, simple blinded study (GAVCA study).

**METHODS:**

In total, 139 eligible patients were enrolled in 9 centers. Catheter placement was evaluated by 3 different components: number of ventricular cannulation attempts, a grading scale, and the anatomical position of the catheter tip. The primary endpoint was the rate of primary cannulation of grade I catheter position in the ipsilateral ventricle. The secondary endpoints were rate of intraventricular position of the catheter's perforations, early ventricular catheter failure, and complications.

**RESULTS:**

The primary endpoint was reached in 70% of the guided group vs 56.5% (freehand group; odds ratio 1.79, 95% confidence interval 0.89-3.61). The primary successful puncture rate was 100% vs 91.3% (*P* = .012). Catheter perforations were located completely inside the ventricle in 81.4% (guided group) and 65.2% (freehand group; odds ratio 2.34, 95% confidence interval 1.07-5.1). No differences occurred in early ventricular catheter failure, complication rate, duration of surgery, or hospital stay.

**CONCLUSION:**

The guided ventricular catheter application proved to be a safe and simple method. The primary endpoint revealed a nonsignificant improvement of optimal catheter placement among the groups. Long-term follow-up is necessary in order to evaluate differences in catheter survival among shunted patients.

**ABBREVIATIONS**
AE: adverse event
AT: as treated
CSF: cerebrospinal fluid
CI: confidence interval
CT: computed tomography
FH: freehand treatment group
FOHR: fronto-occipital horn ratio
FOHWR: fronto-occipital horn width ratio
GAVCA: Guided Application of Ventricular Catheter
GU: guided treatment group
ITT: intention to treat
MRI: magnetic resonance imaging
OR: odds ratio
PP: per protocol
SAE: severe adverse events

Cerebrospinal fluid (CSF) diversion by shunt implantation for hydrocephalus treatment is a common procedure in neurosurgery, intended to re-establish intracranial pressure changes. Since a CSF shunt represents a life-long implant that needs to enable continuous CSF drainage, shunt malfunction remains a significant burden in follow-up.^1,2^ Thus, shunt revision becomes necessary in up to 16% and 12% after 30 d in pediatric and adult patients, respectively, and in up to 39% and 21% after 1 yr.^3-5^ The main reasons for shunt malfunction are obstruction, infection, and malposition.^6-8^ Shunt obstruction might be caused by protein overload due to hemorrhage or infection, or by overdrainage, when paraventricular tissue is aspirated into the ventricular catheter.^7,9-11^ A growing body of literature reports on relevant rates of incorrect freehand ventricular catheter placement, ranging from 12.3% to 44%.^12-18^ An increased shunt failure rate was reported if the ventricular catheter was positioned with insufficient quality with the catheter tip being not optimally placed in the ventricle.^12,14,16^

Thus, it should be the surgeons’ intent to place the perforated part of the ventricular catheter entirely inside the ventricle optimally with no contact to surrounding tissue.^16,19,20^ While freehand placement is performed according to anatomical landmarks,^21^ technical advances such as neuronavigation,^12,18,19,22-25^ ultrasonography,^18,26,27^ and endoscopy^28,29^ have been suggested to improve the quality of catheter placement. In prospective studies, endoscopy failed to demonstrate improved shunt failure rates while ultrasound showed nonsignificant improvement in the quality of catheter position.^26,28^ In cohort studies, neuronavigation was shown to improve precision and the proximal shunt failure rate.^12,18^ It is a consideration that a more simple but reliable technique may be used in more patients for a part of shunt surgery that takes only a few seconds.

In 1985, Ghajar introduced a ventricular catheter guide for the frontal approach, enabling a perpendicular trajectory relative to the skull surface as a simple tool to improve catheter placement.^30^ Different studies have shown that the perpendicular trajectory is only valid in the sagittal plane, however. In the coronal plane, an individual measurement is necessary for optimal planning.^31-33^ Therefore, a guiding tool was introduced in order to apply the individual coronal angulation of the trajectory measured by a mobile health application (mhealth app).^31,34^ A prospective, randomized, 2-armed, multicenter “Guided Application of Ventricular CAtheter” (GAVCA) trial^35^ was initiated to evaluate the validity of the surgeon's intent to place ventricular catheters correctly by first puncture, and determining early shunt failure and complication rates.

## METHODS

### Objective of the Study

The primary objective of the study is to prove the superiority of guided ventricular catheter placement (guided treatment group, GU) vs the standard freehand technique (freehand treatment group, FH) in terms of optimal catheter positioning in the ventricle.

### Study Design

**Figure 1.**
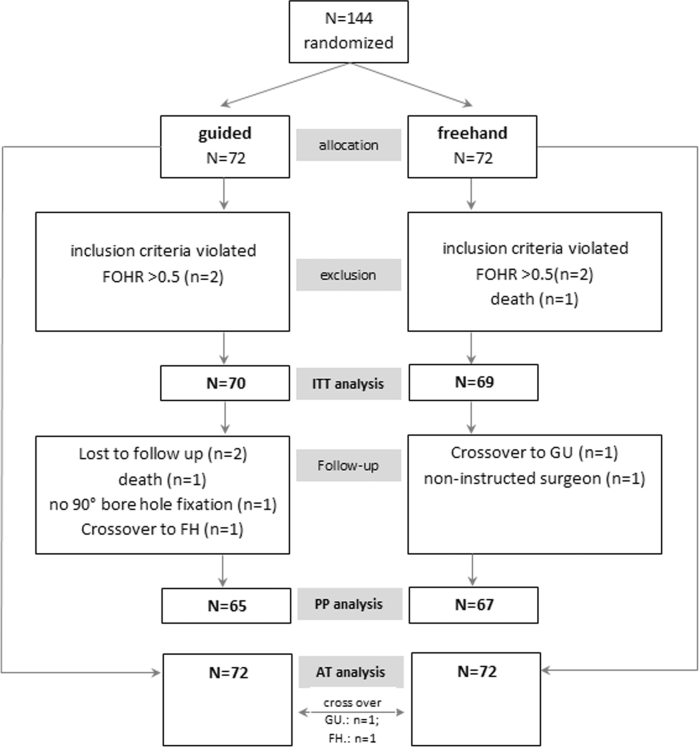
Flow chart of patient population enrolled in the GAVCA study.

**Figure 2.**
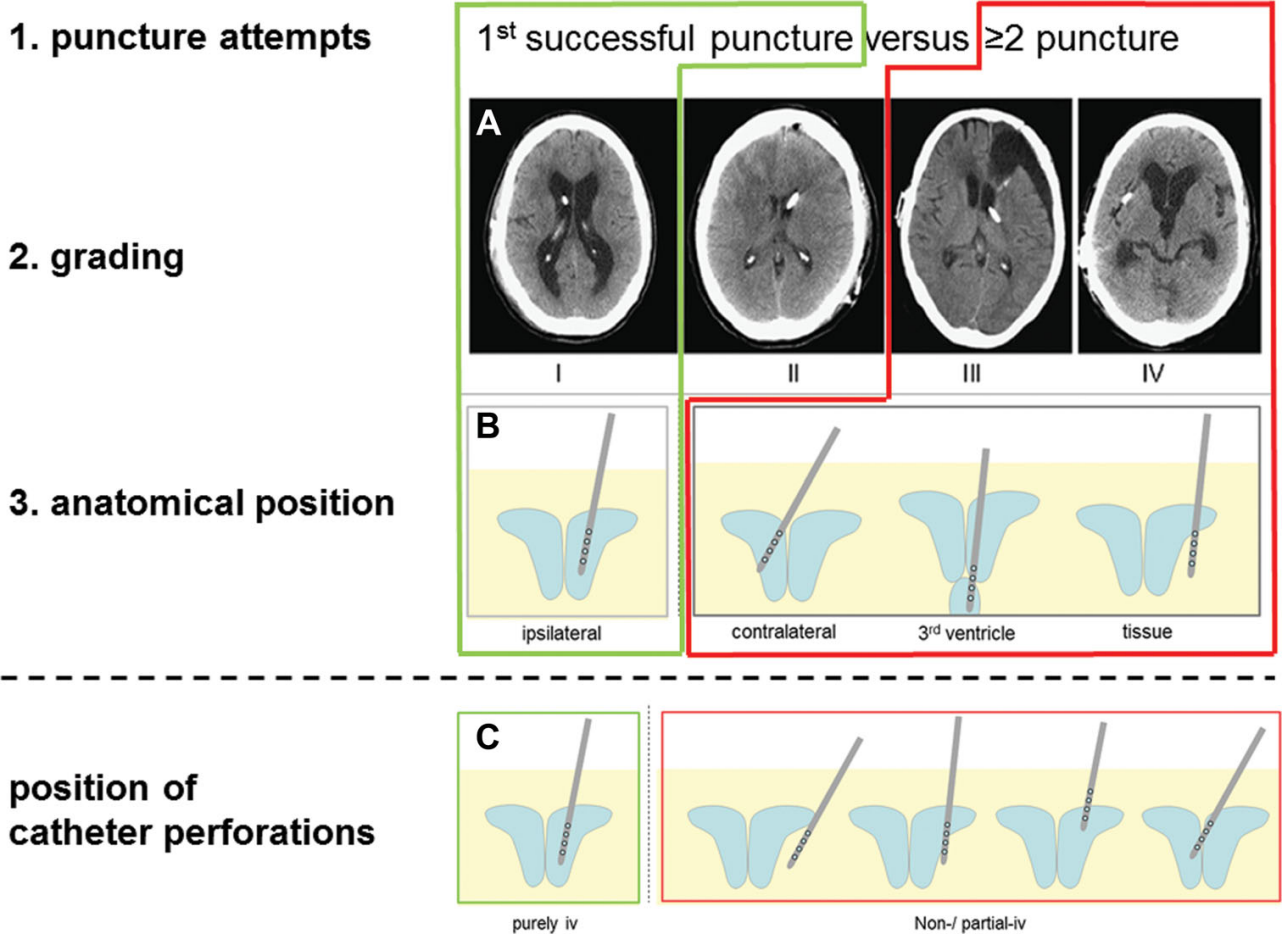
Quality of catheter position was evaluated in different factors. The factors 1 to 3 refer to the primary endpoint analysis combining first, the amount of puncture attempts, second, grading of catheter tip location in relation to paraventricular tissue, and third, anatomical position of the catheter tip. The primary endpoint was defined as an optimal catheter position at first puncture attempt, grade I and ipsilateral ventricle (upper green box). In addition, the incorrect catheter position was defined as ≥2 puncture attempts, grade II and IV, and nonipsilateral catheter position (upper red box). **A**, Grading scale (for distal 2 cm of catheter): grade I: catheter position without contact of more than 0.5 cm to the ventricular wall; grade II: contact of more than 0.5 cm to the ventricular wall or the choroid plexus; grade III: only partially intraventricular position of the catheter tip (less than 1.5 cm intraventricular); grade IV: extraventricular position of the catheter (less than 0.5 cm intraventricular). **B**, Anatomical catheter position with the catheter tip position located in the ipsilateral, contralateral, third ventricle, or in the tissue. **C**, As a secondary endpoint, the rate of complete intraventricular position of the perforated part of the catheter tip was defined. Aside from the complete extraventricular position of the catheter tip, different possible scenarios for perforations being in contact with adjacent brain tissue. That represents either the catheter is positioned too short, too long, has perforated the septum or not is inside the ventricles (iv, intraventricular).

The GAVCA study was designed as a prospective, controlled, randomized, multicenter study and was described previously^35^ and registered at http://clinicaltrials.gov/ct2/show/NCT01811589. The trial protocol was approved by the institutional review boards of the coordinating center (Charité Medical University Centre, Berlin, Germany, EA2/168/12) and all collaborating institutions (Clinical Trial Registration Information NCT Number: NCT01811589). The patients were randomized 1:1 balanced in both arms (Figure 1, see **Supplemental Methods, Supplemental Digital Content**, for further details) with lists prepared for each study center to prevent center-specific effects. The randomization allocation was available from an online password-protected database. An individual randomization plan with random permuted blocks was generated using statistical software (SAS; SAS Institute Inc, Cary, North Carolina). The study was performed in 9 different neurosurgical centers. Among other parameters, after surgery the amount of cannulation attempts were documented until CSF flow sets in. A neuroradiologist was blinded for evaluation of ventricular catheter placement using thin-sliced postoperative CT or MRI data sets (cMRI: volume data sets [MP-Rage, 3D-FFE] and thin-section T2 in 3 planes; cCT: Thin-section spiral CT reconstruction or thin-section CT with 1.25-2 mm sections). The data sets were pseudonymized and sent on a medium to the assigned neuroradiologist for evaluation. Determination of the radiological variables was conducted in 3 different rating systems: first, the catheter tip position in the ventricle being surrounded by CSF using a grading scale (I°-IV°; Figure 2A); second, the anatomical position of the catheter tip (>5 mm of distal end, ipsilateral, contralateral, third ventricle, tissue; Figure 2B); and third, the exact measurements of the perforated catheter tip being completely, intraventricularly positioned (Figure 2C).

The primary endpoint of this study combines 3 aspects of catheter placement: first, the number of cannulation attempts until CSF flow sets in; second, the grading scale; and third, the anatomical position of the catheter tip in postoperative imaging (Figures 2A and 2B). Thus, the primary endpoint was defined as primary successful catheter cannulation as grade I catheter position in the ipsilateral ventricle representing the *optimal* catheter position. In addition, the *incorrect* catheter position is defined post hoc as nonprimary cannulation, grade III and IV and nonipsilateral position.

The secondary endpoints were defined as the percentage of correct intraventricular localizations of the perforated catheter part (Figure 2C), early ventricular catheter failures requiring ventricular catheter revision within 30 d, and complication rates.

### Device and Software Description

**Figure 3.**
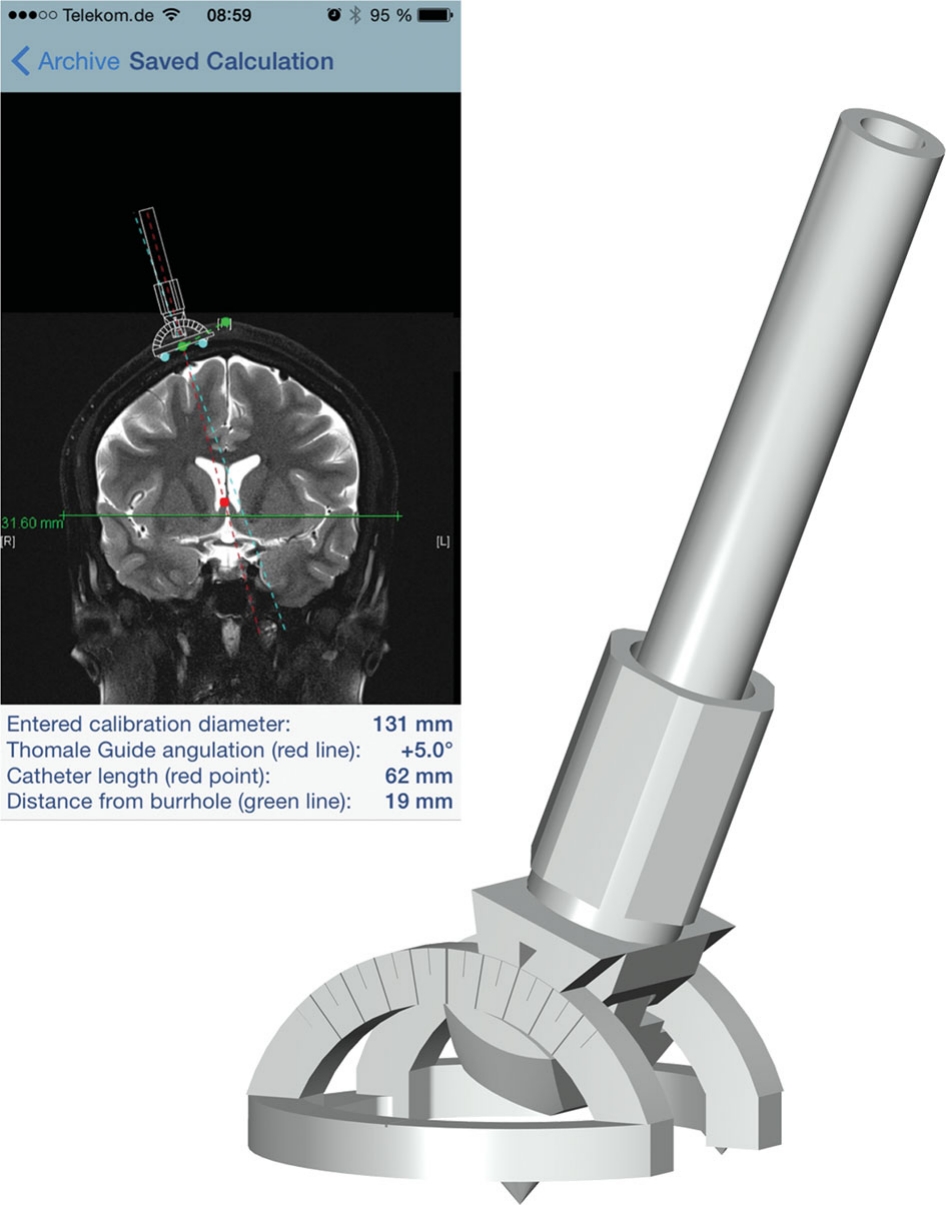
The surgical instrument used as a ventricular catheter guide (**right**) and a screenshot of the mobile health application (**left**) with the measured parameters for correct catheter placement.

The ventricular catheter guide (Thomale Guide, Christoph Miethke GmbH & Co KG, Potsdam, Germany, Figure 3) is a surgical instrument that allows for a rectangular orientation of the trajectory towards the skull in the sagittal plane and the application of an individual angle in the coronal plane.^31^ The mhealth app (iOS, iTunes: Thomale Guide App, Christoph Miethke GmbH & Co KG, Figure 3), was developed to measure the catheter trajectory (angle deviation from perpendicular trajectory to skull surface, catheter length, and entry point's distance to midline) on a previously imported, coronal reconstructed CT or MRI image section.^31,34^ The guide, together with the software application, has been approved for clinical use (EU certificate registration number: 009066 M2R, Christoph Miethke GmbH & Co KG).

### Intervention

CSF shunting in hydrocephalic patients is a standard neurosurgical procedure. The placement of a ventricular catheter is taught at an early stage of neurosurgical residency. The handling of the catheter guide was trained in a standardized workshop held at every center. The entry point used is planned at 10 to 12.5 cm from the nasion and 2 to 3.5 cm from midline. In the freehand-arm anatomical orientation points are used to place the catheter, while in the guided arm the guide is used with the individual parameters of coronal angulation to the skull surface and the catheter length as well as the distance to midline is measured using the dedicated mhealth app.

### Patient Population

**Figure 4.**
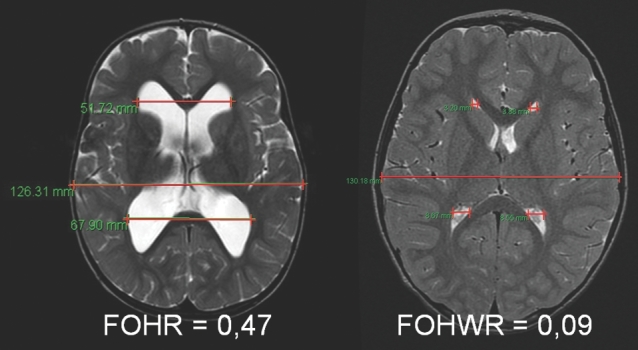
Range of ventricular width for including the patients for this study was FOHR < 0.5 and FOHWR > 0.05. The range of ventricular width is shown on representative MRIs with an FOHR of 0.47 (**right**) in the upper range and FOHWR 0.09 (**left**) in the lower range.

A total of 144 patients, including a 10% drop-out rate, had to be recruited. The patients were consecutively enrolled with the following inclusion criteria: hydrocephalic patients with indication for shunt implantation, patients with a fronto-occipital horn ratio of (FOHR) <0.5 (Figure 4), positioning of the ventricular catheter via a new brain tissue path, precoronal/frontal approach to the ventricle, and signed informed consent. The following exclusion criteria were defined: previously known uneven bone surface at the site of the approach, slit ventricles with a fronto-occipital horn width ratio (FOHWR) <0.05 (Figure 4), participation in other clinical trials with interfering endpoints, patients unable to give informed consent.

### Data Management

For each study center, approval was obtained from the competent ethic committee. Study meetings were held at the outset and after 1 yr in order to report about the study status and clarify any open questions. Data was documented in the password-protected, online database (see **Supplemental Methods, Supplemental Digital Content**, for further details).

### Statistical Hypothesis

The hypothesis evaluated in this study was the rate of primary, grade I catheter position in the ipsilateral ventricle (response rate) being significantly higher in the guided group than in the freehand treatment group. The intention to treat (ITT) analysis was the primary analysis (see **Supplemental Methods, Supplemental Digital Content**, for further details).

### Statistical Analysis Methods

The primary endpoint was analyzed using the Chi-square test, this analysis is considered to be confirmatory. The 2-sided level for the primary analysis was 5%. The logistic regression was used to determine possible influencing factors. The independent samples *t*-test was used for the duration of surgery and hospitalization analysis. Nonparametric data, such as intracerebral bleeding, intracerebral bleed volume, and the number of cannulation attempts with the ventricular catheter, was analyzed using the Mann–Whitney *U*-test. The secondary parameters were evaluated on an explorative basis. All explorative tests were conducted with a 2-sided level of 5%. The statistical package SAS 9.4 (SAS Institute Inc, Cary, North Carolina) was used for analysis.

## RESULTS

Over the recruitment period between 4/2013 and 12/2014, a total of 144 patients were enrolled across the 9 centers with a follow-up within a period of up to 4 mo. One study center was closed prematurely after the enrollment of 3 patients due to insufficient investigator staff. Eight centers routinely terminated the study as previously planned. In the primary ITT analysis (n = 139; Figure 1), 70 patients remained in the guided treatment arm and 69 patients in the freehand treatment arm. For the PP analysis 132 patients (the guided group: n = 65 vs freehand group: n = 67) and for the AT population 139 patients (guided group: n = 70 vs freehand group: n = 69) were included (for further information see **Supplemental Results, Supplemental Digital Content**). Postoperative imaging was performed at a mean of 1.6 ± 1.7 (0-9) d in the guided group and 1.9 ± 1.9 (0-11) d in the freehand group.

### Baseline Characteristics

The baseline characteristics of the study population are given in Table 1. No statistical differences were seen in age, sex distribution, type of diagnosis, or preoperative ventricular width.

**Table 1.** Patient Characteristics and Variables (ITT Population; Values are Given in Either Mean ± Standard Deviation or Absolute Frequency).

		FH	Gu	
		n = 69	N = 70	*P* value
Age	(yr)	62.5 ± 17.9	61.8 ± 16.7	.80
Gender	Female/male	33/36	31/39	.68
Diagnosis (n)	NPH	34	33	.46
	Posthemorrhagic	18	19	
	Tumor related	5	8	
	Congenital	4	4	
	Posttraumatic	1	4	
	Pseudotumor	2	1	
	Postinfectious		1	
	Miscellaneous	5		
Ventricular width preOP	FOHR	0.44 ± 0.04	0.44 ± 0.05	.57
	FOHWR	0.24 ± 0.06	0.25 ± 0.06	.28
Ventricular width postOP	FOHR	0.44 ± 0.05	0.45 ± 0.04	.18
	FOHWR	0.23 ± 0.07	0.25 ± 0.06	.13
Burr hole	Distance from nasion (cm)	11.63 ± 0.73	11.58 ± 0.55	.29
	Distance from midline (cm)	2.79 ± 0.53	2.67 ± 0.51	.16
	Right/left (n)	58/11	55/15	.41
Calculated angle	(°)	7 (co, n = 1)	5.8 ± 3.2 (0-15)	NA
	Medial/0°/lateral (n)	1/-/- (co, n = 1)	54/3/13	
Calculated Catheter length	(cm)	6.3 (co, n = 1)	6.24 ± 1.1	
Catheter type (n)	Miethke	48	54	.27
	Codman Acco-flo	8	8	
	Codman Bactiseal	10	6	
	others	3	2	
Burr hole fixation (n)	Reservoir	34	35	.15
	Deflector	35	30	
	90° connector	0	4	
	Others		1	
Surgeon Experience (n)	1-2 yr resident	19	14	.26
	3-4 yr resident	16	13	
	5-6 yr resident	9	6	
	Certified neurosurgeon	25	37	
Guide experience (n)	1/2 times		11	NA
	3/4 times	1 (co)	10	
	5/6 times		11	
	>6 times		38	
Hospital stay (d)		15.13 ± 12.6	14.66 ± 12.5	.82
Time of surgery (min)		70.62 ± 36	65.89 ± 22.4	.35

FH – freehand; Gu – guided; NPH – normal pressure hydrocephalus; FOHR – frontal occipital horn ratio; FOHWR – fronto occipital horn width ratio; preOP – preoperatively; postOP – postoperatively; co – cross over; NA – nonapplicable.

### Primary Endpoint

**Figure 5.**
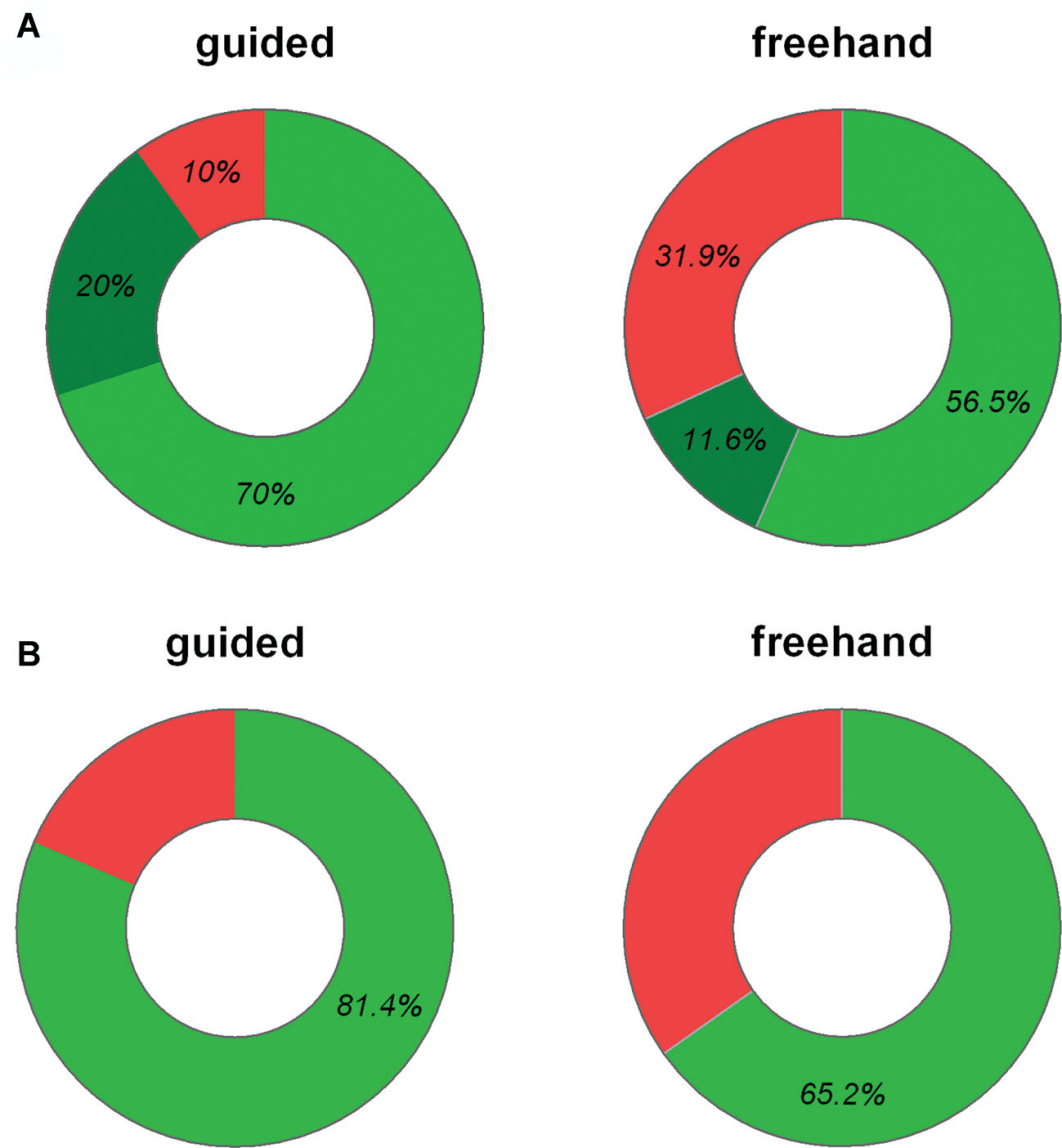
**A**, Rate distribution for the quality of ventricular catheter position in postoperative imaging. The optimal catheter position defined as primary, grade I in the ipsilateral ventricle (light green) reached a rate of 70% in the guided group compared to 56.5% in the freehand group (ITT: *P* = .099; PP: *P* = .137; AT: *P* = .045), while an incorrect catheter position (nonprimary, grade II and IV, nonipsilateral, red) could be avoided significantly more often in the guided group (10% vs 31.9%; *P* = .001). Intermediate catheter position (primary, grade II, ipsilateral; dark green) revealed 20% in the guided and 11.6% in the freehand group. **B**, The rate of complete intraventricular positioning of the catheter perforations was significantly higher in the guided group (81.4% vs 65.2% in the freehand group; *P* = .031).

The primary endpoint of the study is the analysis of the proportion of optimal catheter positioning being primary grade I ventricular catheter tip position in the ipsilateral ventricle. The rate of optimal catheter position was 70% in the guided treatment group vs 56.5% in the freehand treatment group (ITT analysis: *P* = .099; odds ratio [OR] 1.79, 95% confidence interval [CI] 0.89-3.61; PP analysis: *P* = .137; OR 1.72, 95% CI 0.84-3.51; AT analysis: *P* = .045, OR 2.03, 95% CI 1.01-4.12; Figure 5 and Table 2).

**Table 2.** Evaluation of Ventricular Catheter Position (Values are Given in Absolute and Relative Frequencies).

		FH group		GU group			
		%	n	%	n	Test	*P* value
**Combined parameter analysis**							
“Optimal” Primary grade I in il. ventricle	ITT	56.5%	39/69	70%	49/70	Chi-square	.099
	PP	55.9%	38/67	69.7%	45/65		.137
	AT	55.1%	38/69	71.4%	50/70		.045
“Incorrect” Nonprimary, grade III and IV, non-il. ventricle	ITT	31.9%	22/69	10%	7/70	Chi-square	.001
I primary in il. ventricle	ITT	56.5%	39	70	49	*U*-test	.019*
II primary in il. ventricle		11.6%	8	20	14		
III primary, non il. ventricle		23.2%	14	10	7		
IV and nonprimary		8.7%	8	–	–		
**Individual parameter analysis**							
Grading	I	62.3%	43/69	72.8%	51/70	*U*-test	.092*
	II	18.8%	13/69	21.4%	15/70	Chi-square	.184**
	III	15.9%	11/69	5.7%	4/70		.018***
	IV	2.9%	2/69	0%	–		
Anatomical position	il.	84.1%	58/69	94.3%	66/70	*U*-test	.044*
	3^rd^ V	2.9%	2/69	4.3%	3/70	Chi-square	.051^§^
	cl.	7.2%	5/69	0%	–		
	tissue	5.8%	4/69	1.4%	1/70		
Primary puncture	1st	91.3%	63/69	100%	70/70	*U*-test	.012
	2nd	5.8%	4/69				
	3rd	1.5%	1/69				
	4th	1.5%	1/69				
Intraventricular perforations	ITT	65.2%	45/69	81.4%	57/70	Chi-square	.031

il. – ipsilateral; FH – freehand; GU – guided; ITT – intention to treat; PP – per protocol; AT – as treated; 3rd V – third ventricle; cl. – contralateral. *Distribution of grading/anatomical position; **Grade I vs II to IV; ***Grade I and II vs III and IV; ^§^il vs cl, 3rd V, tissue.

### Secondary Endpoints

A successful attempt at catheter placement into the ventricle until CSF flow set in was observed in all cases (100%) in the guided treatment group, and in 91.3% of the freehand treatment group (*P* = .012; *U*-test; Table 2). A complete intraventricular localization of the perforated catheter part was observed more frequently in the guided group (81.4%) than in the freehand treatment group (65.2%; *P* = .031; OR 2.34, 95% CI 1.07-5.1; Table 2).

With regard to possible shunt failure during a follow-up period of 34.2 ± 6.9 d after surgery, a total of 15 operations were performed during the follow-up period. Thirteen were shunt revisions. No differences between the groups were seen as 9 patients in the guided treatment group and 6 patients in the freehand treatment group underwent revision surgery (*P* = .43; Table 3). Among the 3 ventricular catheter revisions that were undertaken, 2 occurred in the guided and 1 in the freehand treatment group (*P* = .57; see **Supplemental Results, Supplemental Digital Content**, for further details).

**Table 3.** Adverse Events, Additional Surgeries, Shunt Revision, Hemorrhage, and Air Inclusion (Values are Given as Absolute Frequency or as Mean ± Standard Deviation).

	FH	GU	*P*
Patients with AE/SAE (n)	22	20	.67
AE (n)	29	21	.14
SAE (n)	19	17	.66
Additional surgeries (n)	6	9	.43
Shunt revisions (n)	4	9	.15
Ventricular catheter revisions (n)	1	2	.57
Hemorrhage (n)	5	6	.77
Hemorrhage volume (cm^3^) (range)	0.55 ± 0.75 (0.003-1.86)	0.71 ± 0.79 (0.075-1.88)	.71
Air inclusion (n)	51	50	.74

AE – adverse events; SAE – severe adverse events.

For the entire cohort of 144 patients, 50 adverse events (AE) were reported for 42 patients (Table 3). Thirty-six AE of the 50 AE were assessed as serious (72%). Severe AE (SAE) were similarly distributed in both groups (see **Supplemental Results, Supplemental Digital Content**, for further details). The evaluation of possible intracerebral hemorrhage or intracranial air signals revealed no differences between the groups (Table 3). Surgery time and duration of hospital stay were similar in both groups (Table 1).

### Post Hoc Analysis of the Factors Included in the Primary Endpoint

Incorrect catheter position, which was defined as grade III-IV or nonprimary placement of the ventricular catheter, amounted to 10% in the guided group vs 31.9% in the freehand group (*P* = .001; OR 4.21, 95% CI 1.66-10.68; Figure 5 and Table 2). Evaluating only the grading scale, a significant difference was seen in a comparison of grades I and II vs III and IV (*P* = .018; OR 3.83; 95% CI 1.18-12.41; Table 2) and between all the groups (*P* = .092, *U*-test). Anatomical positioning of the catheter tip (>5 mm) did differ nonsignificantly when looking at ipsilateral vs other positions (*P* = .051; OR 3.12, 95% CI 0.94-10.36) or differ significantly comparing all different positions between the groups (*P* = .044, *U*-test; Table 2).

### Influencing Factors

Possible influencing factors such as ventricular width, burr hole location, catheter type, burr hole fixation, surgeon training, and guide experience are given in Table 1. No differences in these numbers were seen between the groups.

In a bivariate logistic model, the possible influence of factors with regard to the primary endpoint analysis was evaluated. The factors age, diagnosis, FOHWR, and lateralization of the burr hole did show a level of significance (*P* < .05; Table 4). All factors with a *P* level <.1 were thus included in a multivariate logistic model. Here, age remained the only possible influencing factor on the primary endpoint analysis (Table 4). Excluding the children from the study, the subgroup analysis revealed a rate for optimal catheter positioning of 71% in the guided group vs 58% in the freehand group (*P* = .13; OR 1.72, 95% CI 0.84-3.51), since in 4 children the primary outcome revealed 50% (1/2) in the guided group vs 0% (0/2) in the freehand group. The only factor that reflected a difference between the groups in the primary endpoint was the catheter subgroup, which integrated 0.5 cm marks for controlling the length of insertion (Miethke-catheters). An optimal catheter position rate of 81% (n = 44/54) in the guided group vs 63% (n = 30/48) in the freehand group (*P* = .03, OR 2.64, 95% CI 1.07-6.5) was observed.

**Table 4.** Logistic Regression Analysis.

Factor	*P*
**Bivariate logistic model**	
Center	.11
Age	**.0003**
Sex	.23
Diagnosis	**.0008**
FOHR	**.07**
FOHWR	**.02**
Burr hole lateralization	**.03**
Surgeon experience	.45
Catheter type	**.06**
Type of fixation	.60
**Multivariate logistic model**	
Age	**.025**
Diagnosis	.972
FOHR	.282
FOHWR	.928
Burr hole lateralization	.435
Catheter type	.226
Type of treatment	**.059**

FOHR – frontal occipital horn ratio; FOHWR – frontal occipital horn width ratio.

## DISCUSSION

Obstruction is one of the main causes of CSF shunt malfunction.^9,11,20^ Insufficient placement of the ventricular catheter,^11,13^ the incomplete placement of the ventricular catheter holes inside the ventricle, and repetition of the ventricle cannulation attempts until the CSF flow sets in are stated as surgery-related reasons for shunt obstruction associated with the ventricular catheter.^13,36^ It is the surgeon's intent to place the ventricular catheter tip purely inside the ventricle without contact to adjacent tissue at first attempt, with the goal of reducing shunt failure. Beside other techniques, for the frontal precoronal approach, a possible aim was described to place the catheter tip inside the ipsilateral ventricle superior to the foramen of Monro.^16,19,21,37^

For shunt surgery as well as for external ventricular drains, the freehand technique for ventricular catheters based on anatomical landmarks showed a misplacement rate of 12.3% to 45%.^12,15,17,18,38^ The wide range of malplacement rates may be explained by different surgical techniques but also by the different ways of evaluating incorrect ventricular catheter position. Since the limitation of freehand catheter placement is increasingly acknowledged, different technical advances have been introduced, such as ultrasound,^19,39,40^ neuronavigation^12,18,41^ or guiding instruments.^30,31,42^ The recently introduced ventricular catheter guide for a frontal approach as used in the GAVCA study provides a simplified, individual measure of catheter application and is assisted by an mhealth app. The technique was investigated in a clinical pilot study with 35 patients^31^ and showed accurate results for catheter placement in narrow ventricles.

By investigating primarily the quality of the surgeon's intent to place a ventricular catheter correctly, our current study showed that the guided treatment group achieved optimal, ventricular catheter placement nonsignificantly more often compared to the freehand group (*P* = .099), for hydrocephalus patients undergoing shunt surgery. Nevertheless, the secondary endpoints being the rate of purely intraventricular positioning of the perforated catheter and the primary puncture rate were significantly increased through the use of guided catheter placement. We must state that the study has been slightly underpowered and can hypothesize that the inclusion of more patient numbers may also have shown a significant effect for the primary endpoint.

The fact that in post hoc analysis the incorrect catheter placement was significantly reduced must be interpreted, cautiously, since it was not part of the primary endpoint; however, it was calculated on the same scale as used for the primary endpoint analysis. After having analyzed the data, it seems to become clear that the potential of the device might result more in improvement of the rate of incorrect catheter placement rather than optimal catheter position.

An issue of all aiding methods for ventricular catheter placements is cost and surgery time.^42,43^ No significant difference was seen between the treatment arms in terms of surgery time and hospital stay. Thus, the guide may be successfully used in a large cohort of patients without causing a significant time burden for the surgeon.

That the patient's age is a potential relevant factor in the quality of ventricular catheter placement as seen in this study, has also been described earlier.^17,40^ The median age in the GAVCA study was 66 yr. However, the subgroup analysis with a cutoff at 40 yr showed a similar but nonsignificant difference between the groups with a smaller patient number (*P* = .09). Since there were only 4 children included in the study, no conclusion can be drawn for pediatric patients. Among adults, the precise definition of age population might be an important factor in subsequent studies.^44^ In addition, ventricular width may be a factor since smaller ventricles are more difficult to be targeted correctly,^17^ as was shown by FOHWR in the bivariate regression model. A subgroup analysis for smaller FOHWR (<0.25) showed a nonsignificant difference between the groups for smaller patient numbers (*P* = .08).

Possibly, the most relevant functional factor for the quality of catheter placement is the location of perforation holes completely inside the ventricle. This might avoid hole-sealing by any paraventricular tissue.^8^ The guided procedure did achieve a significantly higher rate of complete intraventricular catheter perforations as compared to the freehand treatment group. Studies with long-term, revision-free ventricular catheter survival should prove the relevance of this factor in further investigations.

### Limitations

One of the limitations in designing the study was that outcome measures for the quality of catheter position are poorly described in the literature. There were only few heterogeneous values for calculating correct rates for optimal catheter position up front, for which most previous studies used a 3- to 4-point grading scale by combining different aspects in the quality of ventricular catheter position.^12-14^ In our study, we introduced a combined scaling system to investigate all possible factors being relevant for correct catheter position, separately. These factors were as follows: first, the number of puncture attempts until CSF flow sets in; second, the catheter tip position being in contact with paraventricular tissue; third, the anatomical catheter tip position; and fourth, the position of the perforations of the ventricular catheter being inside the ventricle. Thereby we have tried to establish a sophisticated data collection system as baseline for future studies, in order to better evaluate the relevant aspects of correct catheter position. Since no data were available for this quality measure, a miscalculation of the effect and of the population size can be well explained.

Surgical experience and guide experience were documented in order to measure their possible influence on ventricular catheter tip placement. The level of overall surgical experience had no significant influence on the grade of ventricular catheter tip placement in this study. Since 2 surgeons often work together to perform a shunt procedure, our data do not address the combined experience of the team, but only of the surgeon who performed the puncture. In this context, it is important to mention that the guide application technique was performed in 8 out of 9 centers without previous clinical training. Hence, most data in this study representing the initial experience using the guide reflect that the technique is relatively simple to use. Experience with using the guide was not reflected in statistical analysis, to influence the primary endpoint in the guided group. Still, among all 7 patients (10%) from the guided group that were graded with incorrect placement, the ventricular catheter was placed in a correct trajectory towards the ipsilateral ventricle but was either too long or too short. In 1 patient, this resulted in the need for a catheter revision. This aspect was also reflected in the catheter type subgroup analysis, in which a catheter with imprinted 0.5 cm scale for insertion length showed a better quality measure in the guided group, and hence, a significant difference in the primary endpoint between the groups. Another limitation of our study, that is always true for surgical studies, is that the surgeon cannot be blinded to the respective treatment.

Relevant shunt revision rates remain a burden for children and adults.^3-5,28,45,46^ As a limitation in our study, we only looked at early shunt failure rate 30 d after surgery, which showed no differences between the groups in terms of shunt surgeries as well as ventricular catheter revisions. The relatively short follow-up was chosen based on our primary outcome parameter being defined as the surgeon's intent to place a catheter optimally in the ipsilateral ventricle, without contact to the ventricular wall, in the first attempt. Within a 30-d timeframe, catheter revisions were performed in 1 patient in each group due to malposition. The overall shunt failure rate of 9.4% with a total of 13 revisions was comparable to other series with 12% early shunt failure rate in adults^3^ and 22% in a mixed population.^12^ As it was described earlier, the long-term interaction of foreign body implants with the central nervous system as represented by astrogliosis and inflammatory response around and within the catheter might significantly contribute to catheter failure during follow-up.^9,47^ Therefore, we would hypothesize that the contact of the catheter tip, namely the perforated part, will be most relevant in that tissue interaction may reach the intraluminal space leading to catheter obstruction at some later time point. That could possibly account for catheter positions of grade III and IV as well as the catheter tip being not entirely placed in the intraventricular space, since the perforated part and thereby the intraluminal space may more likely be exposed to functional relevant tissue interaction.

Finally, the authors would like to emphasize that a conflict of interest as already formally described should be respected. However, the study design to use a blinded neuroradiologist for the endpoint analysis and the detailed description of the collected data was performed in order to possibly overcome this issue and leave room for the reader's own interpretation.

## CONCLUSION

The guided application of ventricular catheters is a safe and simple technique for ventricular catheter placement. The primary endpoint showed a nonsignificant improvement in optimal catheter position. Puncture attempts were successful in all patients, and the ventricular catheter perforations were positioned more often completely inside the ventricle using the guided technique. Long-term evaluation needs to prove a beneficial effect on catheter revision-free survival in further studies.

### Disclosures

Dr Thomale holds a patent on the surgical instrument. All centers received compensation for data documentation from Aesculap AG, Tuttlingen. Dr Thomale, Dr Schaumann, Dr Stockhammer, Dr Lemcke, Dr Schuhmann, Dr Beez, Dr Fritsch, Dr Rohde, and Dr Bohner have received personal fees form B-Braun, Aesculap (Tuttlingen, Germany) in the past.

This study was sponsored and managed by Aesculap AG, Tuttlingen, Germany. The sponsors managed the data collection, data analysis, data interpretation or writing of the report and reviewed the study design as well as the final report. The corresponding authors are responsible for the study design and had complete access to all data obtained in the study and were responsible for writing and submitting the article for publication.

## Supplementary Material

Supplemental File

## Notes

The results of the GAVCA study were presented at the 25th Congress of the European Society for Pediatric Neurosurgery (ESPN) on 11 May 2016 in Paris, France.

## Notes

**Neurosurgery Speaks!** Audio abstracts available for this article at www.neurosurgery-online.com.

## Neurosurgery Speaks (Audio Abstracts)

Listen to audio translations of this paper's abstract into select languages by choosing from one of the selections below.

## References

[1] Stein SC, Guo W Have we made progress in preventing shunt failure? A critical analysis. J Neurosurg Pediatr. 2008;1(1):40-47. 18352802 10.3171/PED-08/01/040

[2] Baird LC, Mazzola CA, Auguste KI Pediatric hydrocephalus: systematic literature review and evidence-based guidelines. Part 5: effect of valve type on cerebrospinal fluid shunt efficacy. J Neurosurg Pediatr. 2014;14(Suppl 1):35-43. 25988781 10.3171/2014.7.PEDS14325

[3] Wu Y, Green NL, Wrensch MR, Zhao S, Gupta N Ventriculoperitoneal shunt complications in California: 1990 to 2000. Neurosurgery. 2007;61(3):557-562; discussion 562-553. 17881969 10.1227/01.NEU.0000290903.07943.AF

[4] Drake JM, Kestle JR, Milner R Randomized trial of cerebrospinal fluid shunt valve design in pediatric hydrocephalus. Neurosurgery. 1998;43(2):294-303; discussion 303-295. 9696082 10.1097/00006123-199808000-00068

[5] Thomale UW, Gebert AF, Haberl H, Schulz M Shunt survival rates by using the adjustable differential pressure valve combined with a gravitational unit (proGAV) in pediatric neurosurgery. Child's Nervous Syst. 2013;29(3):425-431. 10.1007/s00381-012-1956-9 23135777

[6] Browd SR, Ragel BT, Gottfried ON, Kestle JR Failure of cerebrospinal fluid shunts: part I: obstruction and mechanical failure. Pediatr Neurol. 2006;34(2):83-92. 16458818 10.1016/j.pediatrneurol.2005.05.020

[7] Sainte-Rose C, Piatt JH, Renier D Mechanical complications in shunts. Pediatr Neurosurg. 1991;17(1):2-9. 1811706 10.1159/000120557

[8] Sekhar LN, Moossy J, Guthkelch AN Malfunctioning ventriculoperitoneal shunts. Clinical and pathological features. J Neurosurg. 1982;56(3):411-416. 7057239 10.3171/jns.1982.56.3.0411

[9] Harris CA, McAllister JP 2nd What we should know about the cellular and tissue response causing catheter obstruction in the treatment of hydrocephalus. Neurosurgery. 2012;70(6):1589-1601; discussion 1601-1582. 22157548 10.1227/NEU.0b013e318244695f

[10] McGirt MJ, Buck DW, 2nd Sciubba D Adjustable vs set-pressure valves decrease the risk of proximal shunt obstruction in the treatment of pediatric hydrocephalus. Child's Nervous Syst. 2007;23(3):289-295. 10.1007/s00381-006-0226-0 17106749

[11] Tuli S, Drake J, Lawless J, Wigg M, Lamberti-Pasculli M Risk factors for repeated cerebrospinal shunt failures in pediatric patients with hydrocephalus. J Neurosurg. 2000;92(1):31-38. 10616079 10.3171/jns.2000.92.1.0031

[12] Hayhurst C, Beems T, Jenkinson MD Effect of electromagnetic-navigated shunt placement on failure rates: a prospective multicenter study. J Neurosurg. 2010;113(6):1273-1278. 20397892 10.3171/2010.3.JNS091237

[13] Huyette DR, Turnbow BJ, Kaufman C, Vaslow DF, Whiting BB, Oh MY Accuracy of the freehand pass technique for ventriculostomy catheter placement: retrospective assessment using computed tomography scans. J Neurosurg. 2008;108(1):88-91. 18173315 10.3171/JNS/2008/108/01/0088

[14] Janson CG, Romanova LG, Rudser KD, Haines SJ Improvement in clinical outcomes following optimal targeting of brain ventricular catheters with intraoperative imaging. J Neurosurg. 2014;120(3):684-696. 24116721 10.3171/2013.8.JNS13250

[15] Saladino A, White JB, Wijdicks EF, Lanzino G Malplacement of ventricular catheters by neurosurgeons: a single institution experience. Neurocrit Care. 2009;10(2):248-252. 18923816 10.1007/s12028-008-9154-z

[16] Tuli S, O’Hayon B, Drake J, Clarke M, Kestle J Change in ventricular size and effect of ventricular catheter placement in pediatric patients with shunted hydrocephalus. Neurosurgery. 1999;45(6):1329-1333; discussion 1333-1325. 10598700 10.1097/00006123-199912000-00012

[17] Wan KR, Toy JA, Wolfe R, Danks A Factors affecting the accuracy of ventricular catheter placement. J Clin Neurosci. 2011;18(4):485-488. 21256029 10.1016/j.jocn.2010.06.018

[18] Wilson TJ, Stetler WR Jr., Al-Holou WN, Sullivan SE Comparison of the accuracy of ventricular catheter placement using freehand placement, ultrasonic guidance, and stereotactic neuronavigation. J Neurosurg. 2013;119(1):66-70. 23330995 10.3171/2012.11.JNS111384

[19] Nesvick CL, Khan NR, Mehta GU, Klimo P Jr. Image guidance in ventricular cerebrospinal fluid shunt catheter placement: a systematic review and meta-analysis. Neurosurgery. 2015;77(3):321-331; discussion 331. 26103441 10.1227/NEU.0000000000000849

[20] Thomale UW, Hosch H, Koch A Perforation holes in ventricular catheters—is less more? Child's Nervous Syst. 2010;26(6):781-789. 10.1007/s00381-009-1055-8 20024658

[21] Pang D, Grabb PA Accurate placement of coronal ventricular catheter using stereotactic coordinate-guided free-hand passage. Technical note. J Neurosurg. 1994;80(4):750-755. 8151359 10.3171/jns.1994.80.4.0750

[22] Hermann EJ, Capelle HH, Tschan CA, Krauss JK Electromagnetic-guided neuronavigation for safe placement of intraventricular catheters in pediatric neurosurgery. J Neurosurg Pediatr. 2012;10(4):327-333. 22880888 10.3171/2012.7.PEDS11369

[23] Lind CR, Correia JA, Law AJ, Kejriwal R A survey of surgical techniques for catheterising the cerebral lateral ventricles. J Clin Neurosci. 2008;15(8):886-890. 18440818 10.1016/j.jocn.2007.05.013

[24] Reig AS, Stevenson CB, Tulipan NB CT-based, fiducial-free frameless stereotaxy for difficult ventriculoperitoneal shunt insertion: experience in 26 consecutive patients. Stereotact Funct Neurosurg. 2010;88(2):75-80. 20068382 10.1159/000271822

[25] Sampath R, Wadhwa R, Tawfik T, Nanda A, Guthikonda B Stereotactic placement of ventricular catheters: does it affect proximal malfunction rates? Stereotact Funct Neurosurg. 2012;90(2):97-103. 22398576 10.1159/000333831

[26] Whitehead WE, Riva-Cambrin J, Wellons JC 3rd No significant improvement in the rate of accurate ventricular catheter location using ultrasound-guided CSF shunt insertion: a prospective, controlled study by the Hydrocephalus Clinical Research Network. J Neurosurg Pediatr. 2013;12(6):565-574. 24116981 10.3171/2013.9.PEDS1346

[27] Heussinger N, Eyupoglu IY, Ganslandt O, Finzel S, Trollmann R, Jungert J Ultrasound-guided neuronavigation improves safety of ventricular catheter insertion in preterm infants. Brain Dev. 2013;35(10):905-911. 23265618 10.1016/j.braindev.2012.11.008

[28] Kestle JR, Drake JM, Cochrane DD Lack of benefit of endoscopic ventriculoperitoneal shunt insertion: a multicenter randomized trial. J Neurosurg. 2003;98(2):284-290. 12593612 10.3171/jns.2003.98.2.0284

[29] Flannery AM, Duhaime AC, Tamber MS, Kemp J, Pediatric Hydrocephalus Systematic R, Evidence-Based Guidelines Task F Pediatric hydrocephalus: systematic literature review and evidence-based guidelines. Part 3: endoscopic computer-assisted electromagnetic navigation and ultrasonography as technical adjuvants for shunt placement. J Neurosurg Pediatr. 2014;14(Suppl 1):24-29. 25988779 10.3171/2014.7.PEDS14323

[30] Ghajar JB. A guide for ventricular catheter placement. Technical note. J Neurosurg. 1985;63(6):985-986. 4056916 10.3171/jns.1985.63.6.0985

[31] Thomale UW, Knitter T, Schaumann A Smartphone-assisted guide for the placement of ventricular catheters. Child's Nervous Syst. 2013;29(1):131-139. 10.1007/s00381-012-1943-1 23089936

[32] Woo H, Kang DH, Park J Preoperative determination of ventriculostomy trajectory in ventriculoperitoneal shunt surgery using a simple modification of the standard coronal MRI. J Clin Neurosci. 2013;20(12):1754-1758. 24035649 10.1016/j.jocn.2013.01.025

[33] Park J, Son W, Park KS, Kim MY, Lee J Calvarial slope affecting accuracy of Ghajar Guide technique for ventricular catheter placement. J Neurosurg. 2015; 124(5):1429-1433. 10.3171/2015.5.JNS15226 26544778

[34] Thomale UW. Intracranial ventricular catheter placement with a smartphone assisted instrument. Methods Mol Biol. 2015;1256:405-417. 25626554 10.1007/978-1-4939-2172-0_27

[35] Schaumann A, Thomale UW Guided Application of Ventricular Catheters (GAVCA)—multicentre study to compare the ventricular catheter position after use of a catheter guide versus freehand application: study protocol for a randomised trail. Trials. 2013;14:428. 24330776 10.1186/1745-6215-14-428 PMC3866392

[36] Rehman T, Rehman AU, Rehman A A US-based survey on ventriculostomy practices. Clin Neurol Neurosurg. 2012;114(6):651-654. 22257519 10.1016/j.clineuro.2011.12.040

[37] Yamada SM, Kitagawa R, Teramoto A Relationship of the location of the ventricular catheter tip and function of the ventriculoperitoneal shunt. J Clin Neurosci. 2013;20(1):99-101. 22999561 10.1016/j.jocn.2012.01.041

[38] Lind CR, Tsai AM, Lind CJ, Law AJ Ventricular catheter placement accuracy in non-stereotactic shunt surgery for hydrocephalus. J Clin Neurosci. 2009;16(7):918-920. 19329319 10.1016/j.jocn.2008.09.015

[39] Beez T, Sarikaya-Seiwert S, Steiger HJ, Hanggi D Real-time ultrasound guidance for ventricular catheter placement in pediatric cerebrospinal fluid shunts. Child's Nervous Syst. 2015;31(2):235-241. 10.1007/s00381-014-2611-4 25564197

[40] Whitehead WE, Riva-Cambrin J, Wellons JC 3rd Factors associated with ventricular catheter movement and inaccurate catheter location: post hoc analysis of the hydrocephalus clinical research network ultrasound-guided shunt placement study. J Neurosurg Pediatr. 2014;14(2):173-178. 24926971 10.3171/2014.5.PEDS13481

[41] Jung N, Kim D Effect of electromagnetic navigated ventriculoperitoneal shunt placement on failure rates. J Korean Neurosurg Soc. 2013;53(3):150-154. 23634264 10.3340/jkns.2013.53.3.150 PMC3638267

[42] Yamada SM, Yamada S, Goto Y A simple and consistent technique for ventricular catheter insertion using a tripod. Clin Neurol Neurosurg. 2012;114(6):622-626. 22244253 10.1016/j.clineuro.2011.12.025

[43] Kim D, Son W, Park J Guiding protractor for accurate freehand placement of ventricular catheter in ventriculoperitoneal shunting. Acta Neurochir. 2015;157(4):699-702. 25619775 10.1007/s00701-015-2349-0

[44] Sarrafzadeh A, Smoll N, Schaller K Guided (VENTRI-GUIDE) versus freehand ventriculostomy: study protocol for a randomized controlled trial. Trials. 2014;15:478. 25480528 10.1186/1745-6215-15-478 PMC4289205

[45] Lund-Johansen M, Svendsen F, Wester K Shunt failures and complications in adults as related to shunt type, diagnosis, and the experience of the surgeon. Neurosurgery. 1994;35(5):839-844; discussion 844. 7838331 10.1227/00006123-199411000-00006

[46] Lemcke J, Meier U, Muller C Safety and efficacy of gravitational shunt valves in patients with idiopathic normal pressure hydrocephalus: a pragmatic, randomised, open label, multicentre trial (SVASONA). J Neurol, Neurosurg Psychiatry. 2013;84(8):850-857. 23457222 10.1136/jnnp-2012-303936 PMC3717598

[47] VandeVord PJ, Gupta N, Wilson RB Immune reactions associated with silicone-based ventriculo-peritoneal shunt malfunctions in children. Biomaterials. 2004;25(17):3853-3860. 15020161 10.1016/j.biomaterials.2003.10.024

