# Supplementary material for: GAVCA Study: Randomized, Multicenter Trial to Evaluate the Quality of Ventricular Catheter Placement with a Mobile Health Assisted Guidance Technique
Source: Neurosurgery. 2017 Aug 4;83(2):252–62. doi: 10.1093/neuros/nyx420 (PMC6140776; doi:10.1093/neuros/nyx420)
Supplement: Supplemental File [file nyx420_supplemental_file.docx]

**Supplemental digital content**

Methods

*Data Management*

For each study center, approval was obtained from the competent ethic committee. Study meetings were held at the outset and after one year to report on the study status and to clarify any open questions. Data was documented in the password-protected, online database (<http://www.studiesportal.com/>; Aesculap AG, Tuttlingen, Germany). In order to achieve high data quality, the study staff (principal investigator, investigator and study nurse) were trained during study center initiation in respect of the study plan, online CRF and the preparation and surgical use of the guide. Monitoring visits of the study centers and neuroradiologist data analysis as well as central data reviews were performed by the data manager in order to ensure data plausibility and completeness. The correctness of study data, such as informed consent forms, personal data pseudonymization, AE, SAEs and conformity, was checked. Possible discrepancies were documented in either monitoring visit reports or data clarification requests to be resolved by a defined deadline. All requests were followed up until the queries were resolved. Each study center was monitored two to four times by the study manager.

*Intervention*

The surgical procedure starts with preparation of a skin incision at the precoronal paramedian skull surface at recommended measures of 10 to 12.5 cm from nasion and 2 to 3.5 cm from midline, to expose at least 2 cm in diameter of the calvarium. The exposed dura mater is incised and the leptomeninges are coagulated at the entry point. In one treatment arm, the catheter guide is used with the individual parameters of coronal angulation to the skull surface and catheter length is measured in a coronal section of the cranium using the dedicated mhealth app. In the other treatment arm, the standard freehand technique is employed for catheter placement. In both arms, the catheter is fixed in a rectangular fashion from burr hole to the subcutaneous path using a deflector, for instance. If cannulation is unsuccessful in the freehand treatment arm, the surgeon may retry the puncture. In the guided catheter group, the surgeon is supposed to make a second attempt with the guide in case any mistakes in its application are detected; if not, the surgeon may then switch to the freehand technique.

*Randomization and blinding*

The treatment was allocated to patients after written informed consent, preoperatively, by the treating physician. The treating physician was given the randomized allocation from the online database after written informed consent. The patient and the independent clinical evaluator (neuroradiologist), responsible for the assessment of the primary endpoint were not aware of the treatment allocation. Blinding towards treatment (guide usage) was impossible for the treating surgeon.

*Statistical Hypothesis*

The hypothesis evaluated in this study was the rate of primary, grade I catheter position in the ipsilateral ventricle (response rate) being significantly higher in the Guided group (P_GU_) than in the freehand treatment group (P_FH_).

H 0: P_GU_‑P_FH_ < = 0 (P_GU_< = P_FH_); H 1: P_GU_‑P_FH_ > 0 (P_GU_ > P_FH_).

The intention to treat (ITT) analysis was the primary analysis based on the ITT population. The second analysis was based on the per protocol (PP) population. For the PP analysis, the protocol violations not defined in inclusion or exclusion criteria were excluded. The as treated (AT) analysis was based on the actual treatment, recognizing possible crossovers and was used to evaluate the safety parameters (see Supplemental Methods, Supplemental Digital Content for further details).

*Sample Size Estimation*

The sample size calculation was based on the results of a retrospective, mono-center, non-randomized study of 25 post surgery images (35 patients) ^30^. In 92% of the patients, the ventricular catheter was placed successfully in the ipsilateral ventricle. In randomized, controlled, blinded, multi-center studies the effect is usually smaller. Due to the study design, a lower value of 80% was assumed for the study group and 54% for the freehand group.
Furthermore, due to the fact that some of the patients (5%) might have to be excluded due to any violation of study criteria, the rate was reduced to 78.8% (weighted average). The calculation was performed on the basis of a two‑sided Chi‑squared test with a 5% significance level and a power of 80%. 72 patients were to be enrolled in each arm, including a 10% drop‑out rate using STATA 12.1 (StataCorp, College Station, Texas, USA). The freehand group´s positive rate of 54% was based on an adult cohort study ^13^.

Results

Patient population

In the primary ITT analysis (n=139; figure 1), 70 patients remained in the guided treatment arm and 69 patients in the freehand treatment arm. 2 patients were excluded from the guided treatment group because the inclusion criteria were violated by randomizing a patient with FOHR > 0.5. In the freehand treatment group, one patient died before a postoperative image could be obtained and, in the case of two patients, the same inclusion criteria of FOHR >0.5 was violated compared to the guided treatment arm.
For the PP analysis (n=132), five patients were excluded in the guided treatment arm. In the freehand treatment group, two patients were excluded (figure 1). In one patient in each group, the treatment modality was changed. In the guided group, the instrument was not available during surgery and in the freehand group, the center decided to change to guided treatment. Both were analyzed as crossovers in AT population (n=139; guided group: n=70 vs. freehand group: n=69). For safety analysis, all patients, including those who died before reaching the primary endpoint, were included in the safety analysis (guided group: n=72 vs. freehand group n=72). No patient withdrew their consent to participate in the GAVCA study and no other data was missing.

Surgical revisions

With regard to possible shunt failure, a total of 15 reoperations (10.4%) were performed during the follow-up period. 2 surgeries were epidural hematoma evacuations after cranioplasty parallel to shunt implantation. 13 (9%) shunt revisions were performed. 2 patients developed a shunt infection (infection rate: 1.5%), 1 patient had a wound revision. 10 patients had some form of shunt malfunction, including 4 distal peritoneal catheter revisions, 1 distal subcutaneous catheter revision, 2 proximal subcutaneous catheter revisions and 3 ventricular catheter revision. Among the latter, two occurred in the guided and one in the freehand treatment group (p=0.57). In the freehand treatment group, this catheter was graded as extraventricular. In the guided treatment group, 1 catheter was graded with catheter tip location being anatomically intraparenchymal (grade III), which was placed too long; however, the trajectory in the ipsilateral ventricle was in the correct projection. The second catheter revision occurred after secondary catheter occlusion due to delayed intraventricular hemorrhage. The catheter position was evaluated as grade I, ipsilateral ventricle, 1^st^ puncture.
The remaining surgeries were two epidural hematoma surgeries. No differences between the groups were seen as 9 patients in the guided treatment group and 6 patients in the freehand treatment group underwent revision surgery (p=0.43; table 3).

Adverse events

For the entire cohort of 144 patients, 50 AEs were reported for 42 patients (table 3). 36 AEs of the 50 AEs were assessed as serious (72%). SAEs were similarly distributed in both groups.
15 SAEs were documented for additional surgery as described above. 9 AEs were documented for under- and over-drainage (GU: n=2; FH: n=7; 7 serious). Intracranial hematomas were described in 3 patients (GU: n=1, FH: n=2). The same amount and distribution was found for pain and epileptic seizure. Death, wound dehiscence and shunt infection were distributed as 1 event per treatment group. 2 patients (1.4%) died during the study, one as a result of a delayed intracerebral and subsequent subdural hematoma. The event was causally correlated to the surgery but not to the product. The other patient died from a concomitant metastasizing stomach cancer. A total of 21 AEs were not related to surgery (GU: n=6; FH: n=15) including AEs such as corneal ulcer and urinary infection. In total, 29 of the AEs were classified as causally related or possibly causally related to the surgery (GU: n=14; FH: n=15). 1 AE was assessed as having a causal relationship with the ventricular catheter guide medical device. The catheter appeared to be in the correct trajectory but too long in the postoperative CT and was revised. Two further AEs were documented as possibly causally related to the medical device. One patient experienced a generalized tonic clonic seizure but did not show any signs of hemorrhage in the postoperative CT. The other patient suffered from over-drainage.
